# Association of a novel nutritional marker, the triglyceride-cholesterol-body weight index, with 90-day unfavorable outcomes in acute ischemic stroke: a prospective cohort study

**DOI:** 10.3389/fnut.2025.1707231

**Published:** 2026-01-06

**Authors:** Binhui Xiao, Jiaqian Zhu, Yong Han

**Affiliations:** 1 Department of Neurosurgery, Shenzhen Yantian District People's Hospital (Group), Southern University of Science and Technology Yantian Hospital, Shenzhen, Guangdong, China; 2 Department of Neurosurgery, The First Affiliated Hospital of Shenzhen University, Shenzhen Second People's Hospital, Shenzhen University, Shenzhen, Guangdong, China; 3 Department of Emergency, Shenzhen Second People's Hospital, The First Affiliated Hospital of Shenzhen University, Shenzhen, Guangdong, China

**Keywords:** acute ischemic stroke, modified Rankin scale score, non-linear relationships, prognosis, triglyceride-total cholesterol-bodyweight index (TCBI)

## Abstract

**Objective:**

Currently, research on the relationship between triglyceride-total cholesterol-body weight index (TCBI) and prognosis in patients with Acute Ischemic Stroke (AIS) is relatively limited. Therefore, this study aims to investigate the association between TCBI and the incidence of unfavorable functional outcomes in AIS patients.

**Methods:**

This secondary analysis included 1,764 AIS patients admitted to a South Korean hospital from January 2010 to December 2016. Binary logistic regression assessed the association between TCBI and 90-day unfavorable outcomes (defined as mRS score ≥3). A logistic regression model with restricted cubic spline functions was employed to explore the potential nonlinear relationship between them. A series of sensitivity analyses (including analyses restricted to participants without DM, without smoking, or with BMI < 28 kg/m^2^, as well as analyses using generalized additive models and E-value calculations) and subgroup assessments were conducted to further validate the robustness of the findings.

**Results:**

After adjusting for confounders, TCBI (per 100-unit increment) was inversely associated with 90-day unfavorable outcomes (OR = 0.972, 95% CI: 0.953–0.991). A nonlinear relationship was identified with an inflection point at TCBI = 1227.3. Below this inflection point, every 100-unit increment in TCBI demonstrated an OR of 0.928 (95% CI: 0.8866–0.9714) for unfavorable outcomes at 90 days. Above this turning point, the corresponding OR was 0.983 (95% CI: 0.955–1.287). Sensitivity analyses showed that the OR (95% CI) for the association between TCBI (per 100-unit) and 90-day unfavorable outcomes were 0.969 (0.944–0.993), 0.971 (0.946–0.996), and 0.972 (0.952–0.992) in participants without DM, without smoking, and with BMI < 28 kg/m^2^, respectively. No significant interactions were observed in subgroup analyses stratified by age, sex, previous stroke/TIA, hypertension, CHD, stroke etiology, or AF (all P for interaction ≥0.05).

**Conclusion:**

This study revealed an independent negative link between TCBI and unfavorable outcomes at 90 days in patients with AIS. A nonlinear relationship between them was observed. When TCBI values were below 1227.3, a significant negative correlation was found. This provides new insights for prognostic stratification, optimization of rehabilitation strategies, and clinical management in AIS patients.

## Introduction

Acute ischemic stroke (AIS) remains a leading cause of disability and death worldwide, posing significant challenges to society and the economy (1, 2). Despite significant advancements in early intervention and rehabilitation in recent years, accurately predicting the neurological prognosis of AIS patients remains challenging (3). The identification and application of reliable prognostic indicators are crucial for effective risk stratification, developing personalized treatment strategies, and improving patient outcomes (4). Currently, age, hypertension, stroke etiology, and diabetes mellitus (DM) are widely regarded as the main prognostic factors for AIS (5–7).

Malnutrition is closely associated with adverse prognoses in various disease states, including heart failure, malignancies, and fractures (8–11). In stroke survivors, malnutrition is a common condition resulting from complex interactions such as comorbidities and swallowing difficulties, with an incidence ranging from 3 to 87% (12). Previous studies have confirmed a significant correlation between the nutritional status of stroke patients at the time of admission and subsequent clinical outcomes (13–15). In recent years, a composite nutritional index that integrates triglycerides (TG), total cholesterol (TC), and body weight (BW), known as the triglyceride-total cholesterol-body weight index (TCBI), has demonstrated important predictive value for various diseases, including heart failure, coronary artery disease (CHD), dilated cardiomyopathy, and associated complications (16–18). Given the impact of malnutrition on the prognosis of AIS patients, we hypothesize that there may be an association between TCBI and the prognosis of AIS patients.

Regrettably, current research on the relationship between TCBI and stroke prognosis is limited and inconsistent. In a study based on data from the Third China National Stroke Registry (CNSR-III), a multivariable model involving 9,708 AIS patients showed that the lowest levels of TCBI are significantly associated with an increased risk of poor functional outcomes (Modified Rankin Scale, mRS score ≥ 3) and recurrent stroke after 1 year, compared to the highest levels of TCBI (19). However, another study involving 299 AIS patients who received intravenous tissue plasminogen activator (IV-tPA) treatment found no significant difference in TCBI levels between the groups with good outcomes (mRS score: 0–2) and poor outcomes (mRS score: 3–6) 3 months after AIS (20). Additionally, previous studies lacked sensitivity analyses in specific populations and systematic subgroup analyses. Moreover, no studies have explored the potential nonlinear dose–response relationship between TCBI and AIS prognosis, which may provide more nuanced guidance for clinical risk stratification. Furthermore, existing studies showed heterogeneity in study populations, sample sizes, adjusted covariates, and outcome definitions. Given these limitations, the relationship between TCBI and short-term prognosis in AIS patients remains unclear. We hypothesize that TCBI is independently and inversely associated with 90-day unfavorable outcomes in AIS patients and that this association may exhibit a nonlinear dose–response pattern. To test this hypothesis, we conducted a secondary analysis using data from a Korean cohort, aiming to clarify the strength of association and dose–response relationship between TCBI and stroke prognosis, thereby providing evidence for improving stroke rehabilitation and management.

## Methods

### Study design

We performed this prospective cohort study utilizing a monocentric registry database from South Korea, incorporating patient data spanning the period between January 2010 and December 2016 (21). The independent variable of interest in this study was TCBI. The dependent variable was the 90-day outcome of patients with AIS, categorized as a binary variable: unfavorable outcome and favorable outcome.

### Data source

The data used in this study was sourced from the article by Kang et al.: “Geriatric nutritional risk index predicts poor outcomes in patients with acute ischemic stroke—Automated undernutrition screen tool” (21). The present work is published under Creative Commons Attribution licensing terms, enabling unrestricted utilization, dissemination, and duplication across all media formats, contingent upon appropriate acknowledgment of the original contributors and source material (21). We extend our appreciation to the data providers for making their research dataset accessible for this analysis.

### Study population

Data collection encompassed Korean AIS patients presenting for hospitalization within a seven-day window following symptom manifestation (21). Information was derived from a monocentric perspective enrollment database. Ethical clearance for the primary investigation was obtained from Seoul National University Hospital’s Institutional Review Board, with patient informed consent requirements being waived (IRB reference: 1009-062-332) (21). Therefore, our secondary analysis was exempt from additional ethical clearance. Furthermore, the primary research adhered to Helsinki Declaration principles, with all methodological procedures implemented according to established guidelines and regulatory frameworks, as documented in the declaration statement (21). Our secondary investigation maintained adherence to identical ethical standards.

**Figure 1:**
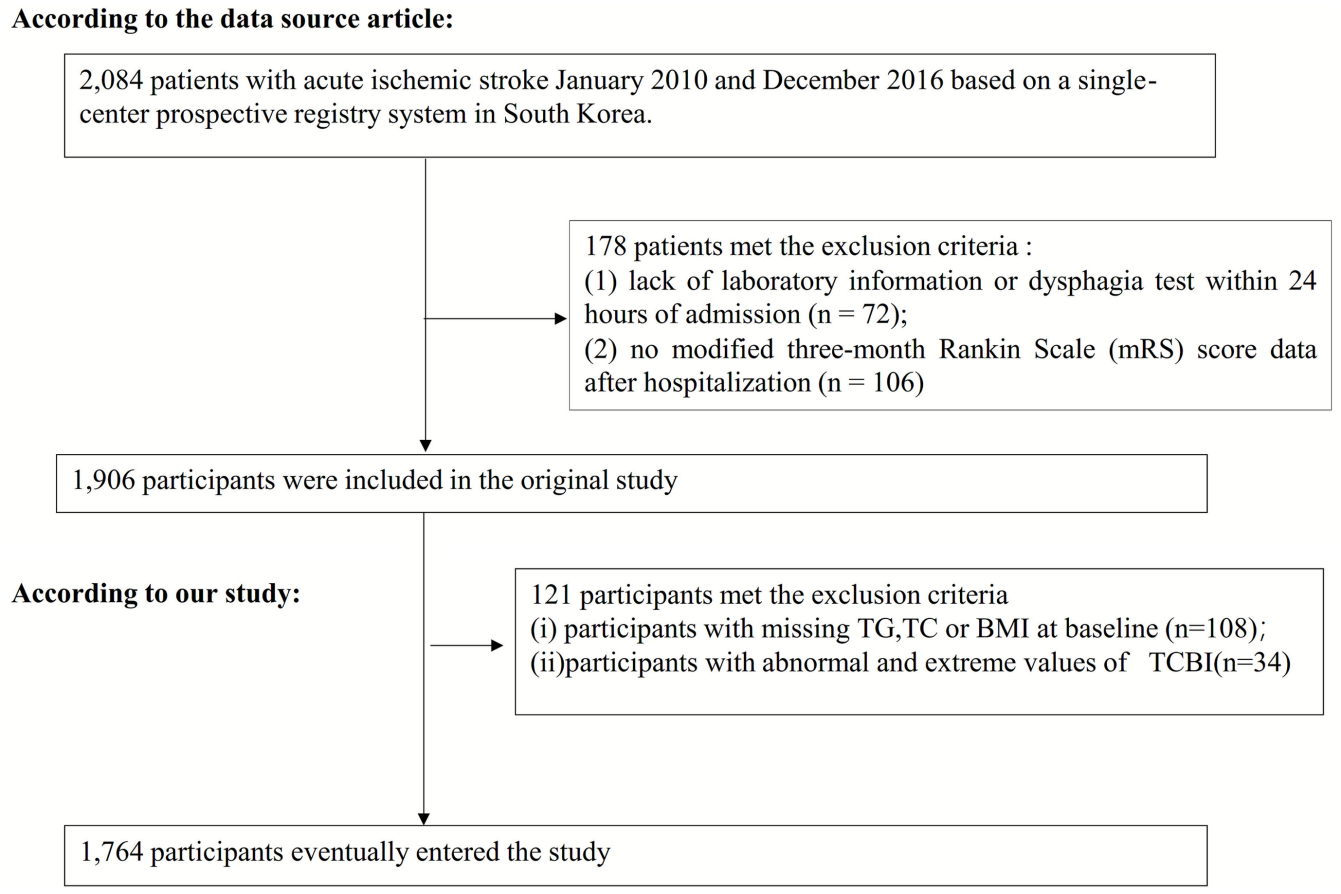
Flowchart illustrating the study participants.

Initially, the original study included 2,084 Korean participants. After excluding 178 participants, a total of 1,906 participants entered the data analysis phase (21). This study further excluded individuals with missing data for TG, TC, and weight (*n* = 108), as well as those with abnormal and extreme TCBI values (defined as deviations greater than or equal to 3 standard deviations from the mean; *n* = 34) (4). Finally, the secondary analysis included 1,764 subjects. Figure 1 presents the selection procedures.

### Variables

The formula for calculating TCBI was (TG × TC × BW)/1,000, where TG and TC were measured in mg/dL, and BW was measured in kg (19).

### Assessment of clinical outcome

Patients were assessed at 90 days after the onset of AIS through outpatient follow-up or structured telephone interviews. The mRS score assessed functional outcomes (21, 22). The primary endpoint of this study was the neurological outcome at 90 days, which was categorized as unfavorable outcome (mRS ≥ 3) and favorable outcome (mRS ≤ 2) (21, 22).

### Covariates

In this study, the selection of covariates was based on previous literature and our clinical experience (4, 23, 24). The following variables were included as covariates: (1) Continuous variables: Hemoglobin A1c (HBA1c), Platelet (PLT), Aspartate Aminotransferase (AST), Nutritional Risk Index (NRI), Height, Low-Density Lipoprotein Cholesterol (LDL-c), Fibrinogen (FIB), Red Cell Distribution Width (RDW), National Institutes of Health Stroke Scale (NIHSS) score, Blood Urea Nitrogen (BUN), High-Sensitivity C-Reactive Protein (Hs-CRP), TC, Albumin (ALB), White Blood Cell (WBC), Estimated Glomerular Filtration Rate (eGFR), BW, Hemoglobin (HGB), TG, Fasting Plasma Glucose (FPG), Alanine Aminotransferase (ALT), High-Density Lipoprotein Cholesterol (HDL-c), and Serum Creatinine (Scr). (2) Categorical variables: Sex, Diabetes Mellitus (DM), previous stroke/transient ischemic attack (TIA), Hypertension, Atrial Fibrillation (AF), Age, Stroke etiology, Smoking, and CHD.

### Missing data processing

Missing data was a common and unavoidable issue in observational studies (25). In the secondary analysis, multiple variables had missing values, including WBC (1, 0.0005%), eGFR (9, 0.005%), ALT (1, 0.0005%), LDL-c (4, 0.002%), FIB (17, 0.009%), NIHSS score (204, 0.116%), FPG (76, 0.043%), Hs-CRP (237, 0.134%), and HBA1c (321, 0.182%). To minimize potential bias from missing data, multiple imputations by chained equations were employed to fill in the missing values in the dataset (25, 26). This approach was based on linear regression and was performed over 10 iterations, assuming that data were missing at random (MAR) (25). The following variables were included in the multiple imputation model: WBC, HGB, RDW, PLT, HDL-c, LDL-c, BUN, Scr, eGFR, AST, ALT, ALB, NIHSS score, FPG, Age, Sex, Previous stroke/TIA, HBA1c, Stroke etiology, Height, Hs-CRP, FIB, TIA, AF, and DM. It should be noted that five imputed datasets were generated in this study. Statistical measures such as means and regression coefficients were calculated separately for each dataset, and these estimates were then pooled using Rubin’s rules.

### Statistical analysis

Subjects were stratified according to TCBI quartile distributions. For continuous measures, normally distributed values were summarized using mean ± standard deviation (SD), while skewed data were presented as median (interquartile range). Categorical data were documented as counts and percentages. We employed the χ^2^ test for categorical variables, conducted one-way analysis of variance (ANOVA) for normally distributed data, and applied the Kruskal-Wallis H test for skewed data to assess the differences among the various TCBI groups.

Three different models were constructed using univariable and multivariable binary logistic regression models to explore the association between TCBI and unfavorable outcomes at 90 days in AIS patients. Model I remained unadjusted for covariates; Model II accounted for demographic variables, including age and sex; Model III was adjusted for DM, PLT, sex, AST, age, HTN, HBA1c, stroke etiology, NIHSS score, HDL-c, AF, smoking, and Scr.

To assess the possible non-linear association between TCBI and 90-day unfavorable outcomes, a logistic regression model with restricted cubic splines was applied. This analysis adjusted for multiple covariates, including DM, PLT, sex, AST, HTN, HBA1c, stroke etiology, NIHSS score, HDL-c, AF, smoking, and Scr. When a non-linear association was detected, inflection points were determined using a recursive method, and separate binary logistic regression models were constructed on either side of the inflection points. Ultimately, the model that best reflected this association was selected through the likelihood ratio test.

Previous studies indicated that obesity, DM, and smoking were closely related to stroke outcomes (27–29). Multiple sensitivity analyses were performed to strengthen the reliability of our results. First, the analysis was limited to individuals with a BMI below 28 kg/m^2^. Subsequently, participants without DM or smoking were included to further assess the stability of our results. Additionally, a generalized additive model (GAM) was employed to incorporate continuous variables as curves into the logistic regression model. Furthermore, to address potential bias introduced during the multiple imputation process, we conducted a complete-case (before multiple imputation) sensitivity analysis by excluding participants with any missing covariates and reassessed the association between TCBI and 90-day unfavorable outcomes in AIS patients. Moreover, given that the exclusion of participants with abnormal and extreme TCBI values (≥ 3 standard deviations from the mean) might have reduced the representativeness of the study population and underestimated the prognostic impact of very low or very high TCBI levels, a sensitivity analysis was conducted in which all participants were included without trimming extreme TCBI values. To further explore the potential impact of unmeasured and unknown confounding factors on the association between TCBI and unfavorable outcomes at 90 days, the E-value was calculated (30).

A stratified binary logistic regression model was used for subgroup analyses based on age, sex, previous stroke/TIA, hypertension, CHD, stroke etiology, and AF. In this analysis, continuous variables such as age were categorized based on clinically relevant cut-offs. Specifically, age was divided into four groups: 80 years and older, 70–80 years, 60–70 years, and under 60 years (31). During the analysis, adjustments were made for DM, PLT, sex, AST, age, HTN, HBA1c, stroke etiology, NIHSS score, HDL-c, AF, smoking, and Scr, while the stratification variables themselves were not included in the adjustments. The log-likelihood ratio test was conducted to compare models with interaction terms and those without, to explore potential interactions.

The receiver operating characteristic (ROC) curve was constructed to evaluate the ability of TCBI to predict 90-day unfavorable outcomes in patients with AIS, and was compared with other nutritional indicators, including TG, ALB, BMI, TC, and NRI. The DeLong test was used to assess the statistical significance of differences between ROC curves. The corresponding area under the curve (AUC), optimal cutoff value, sensitivity, and specificity were also calculated.

Data analyses were performed using R (v3.4.3) and Empower (v4.2) statistical packages. Statistical significance was defined as a two-sided *p*-value < 0.05.

## Results

### Characteristics of participants

**Figure 2:**
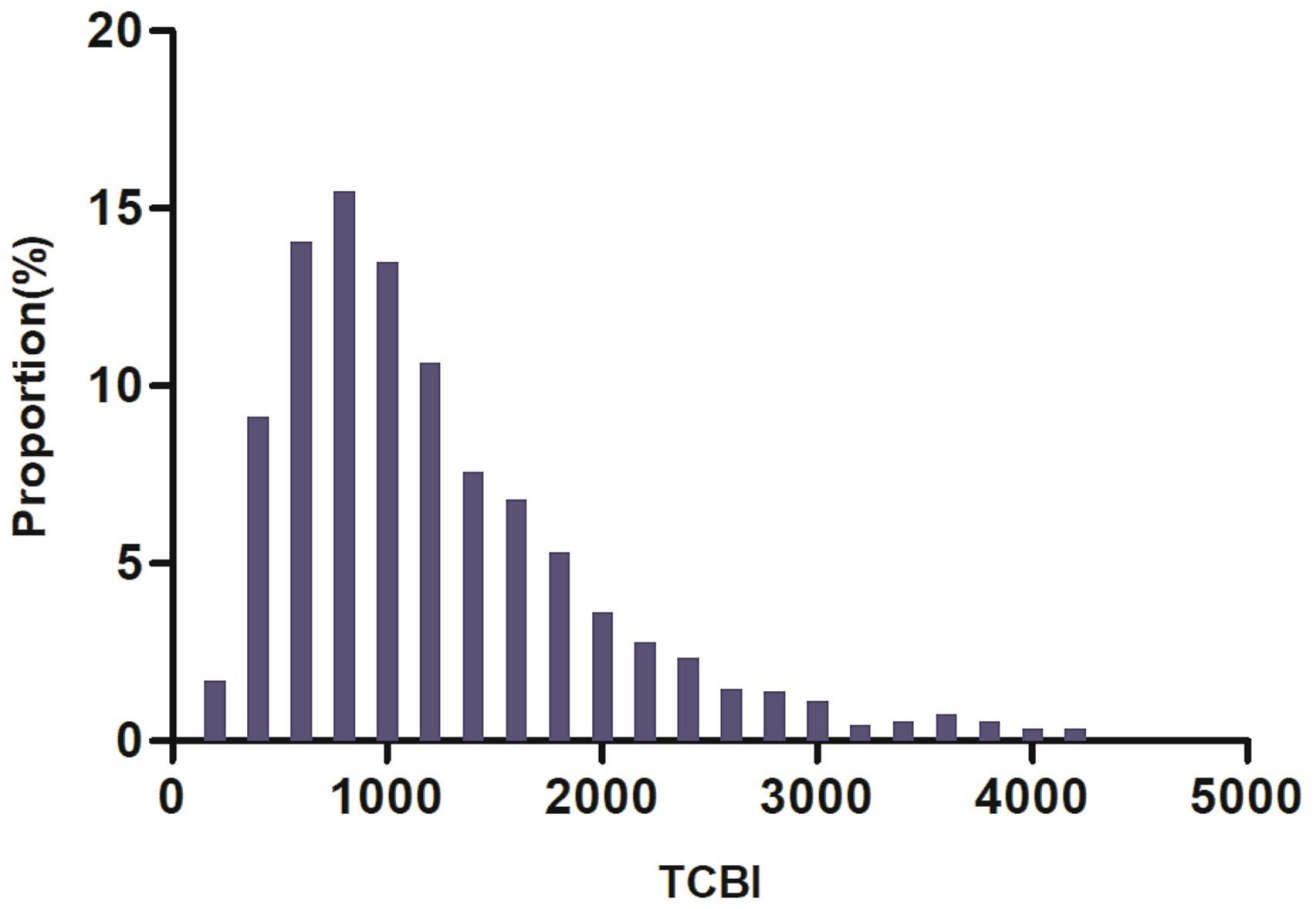
Distribution of TCBI. The distribution appeared skewed, ranging from 186.73 to 4826.73, with a median of 1,051.

The demographic and clinical features of enrolled subjects are summarized in Table 1. A total of 1,764 participants were analyzed, of which 60.77% were male. The TCBI scores exhibited a skewed distribution, ranging from 186.73 to 4826.73, with a median of 1,051 (Figure 2). Based on the TCBI quartiles, the participants were divided into four groups: Q1 (≤703.03), Q2 (703.03–1050.40), Q3 (1051.70–1562.98), and Q4 (>1562.98). Compared to Q1, higher quartile groups had higher levels of PLT, NRI, weight, TC, height, ALT, ALB, HGB, TG, and LDL-c, while levels of RDW, HDL-c, and NIHSS score were lower. Additionally, compared to the lowest quartile group of TCBI, a higher proportion of males and smokers was observed in the higher quartile groups.

**Table 1:** The baseline characteristics of participants.

TCBI quartiles	Q1 (≤703.03)	Q2 (703.03–1050.40)	Q3 (1051.70–1562.98)	Q4 (>1562.98)	*p*-value
Participants (N)	441	441	441	441	
WBC (10^9^/L, mean ±SD)	7.96 ± 3.19	8.17 ± 3.05	8.05 ± 2.77	8.26 ± 2.55	0.454
HGB (g/L, mean ±SD)	12.67 ± 1.97	13.27 ± 1.98	13.69 ± 1.76	14.27 ± 1.81	<0.001
RDW (%, mean ±SD)	13.78 ± 2.08	13.46 ± 1.42	13.25 ± 1.24	13.12 ± 1.13	<0.001
PLT (109/L, mean ±SD)	210.65 ± 72.89	219.55 ± 71.05	228.72 ± 68.58	238.22 ± 69.64	<0.001
TC (mg/dL, mean ±SD)	146.17 ± 32.21	171.38 ± 33.17	188.28 ± 34.41	209.50 ± 41.14	<0.001
TG (mg/dL, mean ±SD)	64.98 ± 16.82	87.58 ± 17.90	110.75 ± 26.51	164.78 ± 48.53	<0.001
HDL-c (mg/dL, mean ±SD)	49.27 ± 14.41	47.84 ± 13.81	47.00 ± 13.31	43.08 ± 12.05	<0.001
LDL-c (mg/dL, mean ±SD)	82.01 ± 26.30	103.37 ± 29.83	116.77 ± 31.62	129.67 ± 39.64	<0.001
BUN (ummol/L, mean ±SD)	18.00 ± 8.42	17.29 ± 9.03	17.84 ± 9.48	16.87 ± 8.09	0.199
Scr (umol/L, median quartile)	0.87 (0.72–1.06)	0.86 (0.73–1.05)	0.91 (0.74–1.08)	0.90 (0.76–1.10)	0.878
NRI	99.87 ± 8.80	103.25 ± 9.15	107.12 ± 8.00	110.05 ± 8.54	<0.001
eGFR (mL/min/1.73 m^2^, median quartile)	78.68 ± 28.01	80.27 ± 29.08	76.56 ± 24.83	78.92 ± 23.78	0.219
AST (U/L, mean ±SD)	26.10 ± 10.82	26.73 ± 14.61	25.55 ± 16.64	24.76 ± 9.65	0.151
ALT (U/L, mean ±SD)	20.70 ± 14.04	21.98 ± 17.30	21.72 ± 14.59	23.89 ± 15.73	0.021
ALB (g/dL)	3.90 ± 0.43	3.96 ± 0.43	4.09 ± 0.39	4.14 ± 0.37	<0.001
BW (kg)	56.11 ± 9.38	60.33 ± 9.33	64.19 ± 9.91	68.45 ± 10.58	<0.001
NIHSS score (mean ±SD)	7.18 ± 6.11	6.95 ± 6.27	5.13 ± 4.82	4.86 ± 5.18	<0.001
FPG (mg/dL, mean ±SD)	105.42 ± 37.26	106.78 ± 40.16	105.19 ± 33.51	108.81 ± 38.78	0.458
Height (cm, mean ±SD)	160.78 ± 8.80	162.03 ± 8.30	163.41 ± 8.20	164.81 ± 8.66	<0.001
HBA1C (%, mean ±SD)	6.20 ± 1.07	6.14 ± 0.99	6.31 ± 1.09	6.41 ± 1.26	0.002
Hs-CRP (mg/L,median quartile)	0.20 (0.05–0.82)	0.22 (0.07–1.24)	0.13 (0.05–0.64)	0.15 (0.06–0.47)	<0.001
FIB (mg/dL, mean ±SD)	331.09 ± 81.80	338.68 ± 90.10	332.43 ± 86.28	332.61 ± 79.91	0.548
Age (years, mean ±SD)					<0.001
<60	58 (13.15%)	84 (19.05%)	98 (22.22%)	146 (33.11%)	
60–70	92 (20.86%)	119 (26.98%)	119 (26.98%)	140 (31.75%)	
70–80	182 (41.27%)	167 (37.87%)	162 (36.73%)	119 (26.98%)	
≥80	109 (24.72%)	71 (16.10%)	62 (14.06%)	36 (8.16%)	
Sex					<0.001
Male	242 (54.88%)	253 (57.37%)	276 (62.59%)	301 (68.25%)	
Female	199 (45.12%)	188 (42.63%)	165 (37.41%)	140 (31.75%)	
Previous stroke/TIA (n, %)	112 (25.40%)	100 (22.68%)	95 (21.54%)	61 (13.83%)	<0.001
CHD (n, %)	61 (13.83%)	56 (12.70%)	51 (11.56%)	39 (8.84%)	0.120
Stroke etiology (n, %)					<0.001
SVO	126 (28.57%)	138 (31.29%)	148 (33.56%)	160 (36.28%)	
LAA	61 (13.83%)	72 (16.33%)	95 (21.54%)	115 (26.08%)	
CE	158 (35.83%)	125 (28.34%)	95 (21.54%)	72 (16.33%)	
Other determined	36 (8.16%)	40 (9.07%)	41 (9.30%)	32 (7.26%)	
Undetermined	60 (13.61%)	66 (14.97%)	62 (14.06%)	62 (14.06%)	
AF (n, %)	135 (30.61%)	104 (23.58%)	84 (19.05%)	53 (12.02%)	<0.001
DM (n, %)	141 (31.97%)	129 (29.25%)	147 (33.33%)	141 (31.97%)	0.616
Smoking (n, %)	135 (30.61%)	156 (35.37%)	187 (42.40%)	219 (49.66%)	<0.001
Hypertension (n, %)	271 (61.45%)	274 (62.13%)	288 (65.31%)	287 (65.08%)	0.522

ALB, albumin; ALT, alanine aminotransferase; AST, aspartate aminotransferase; AF, atrial fibrillation; BUN, blood urea nitrogen; BW, body weight; CE, Cardiac Embolism; CHD, Coronary Heart Disease; DM, diabetes mellitus; eGFR, estimated glomerular filtration rate; FPG, fasting plasma glucose; FIB, fibrinogen; HGB, hemoglobin; HBA1c, hemoglobin A1c; HDL-c, high-density lipoprotein cholesterol; Hs-CRP, high-sensitivity C-reactive protein; LAA, Large Artery Atherosclerosis; LDL-c, low-density lipoprotein cholesterol; NIHSS, National Institutes of Health Stroke Scale score; PLT, platelets; RDW, red cell distribution width; Scr, serum creatinine; SVO, Small Vessel Occlusion; TC, total cholesterol; TG, triglycerides; TIA, transient ischemic attack; WBC, white blood cell; NRI, nutritional risk index.

### Incidence of unfavorable outcomes 90-day in AIS patients

Table 2 summarized the incidence of unfavorable outcomes within 90 days in patients with AIS. In this study, 496 participants experienced unfavorable outcomes, corresponding to an overall incidence rate of 28.12%. In the TCBI quartile groups, the incidence rates of unfavorable outcomes from the Q1 group to the Q4 group were 36.28, 32.14, 23.13, and 18.82%, respectively. Notably, compared to the Q1 group, the incidence rate of unfavorable outcomes at 90 days in the Q4 group was significantly lower (P for trend < 0.001).

**Table 2:** Incidence rate of unfavorable outcome (mRS score ≥3) 90-day after acute ischemic stroke (%).

TCBIquartiles	Participants	unfavorable outcome (events)	Incidence of unfavorable
Total	1764	496	28.12 (26.12–30.22)
Q1	441	160	36.28 (31.78–40.79)
Q2	441	151	32.14 (29.79–38.69)
Q3	441	102	23.13 (19.18–27.08)
Q4	441	83	18.82 (15.16–22.48)
P for trend			<0.001

mRS, modified Rankin scale.

**Figure 3:**
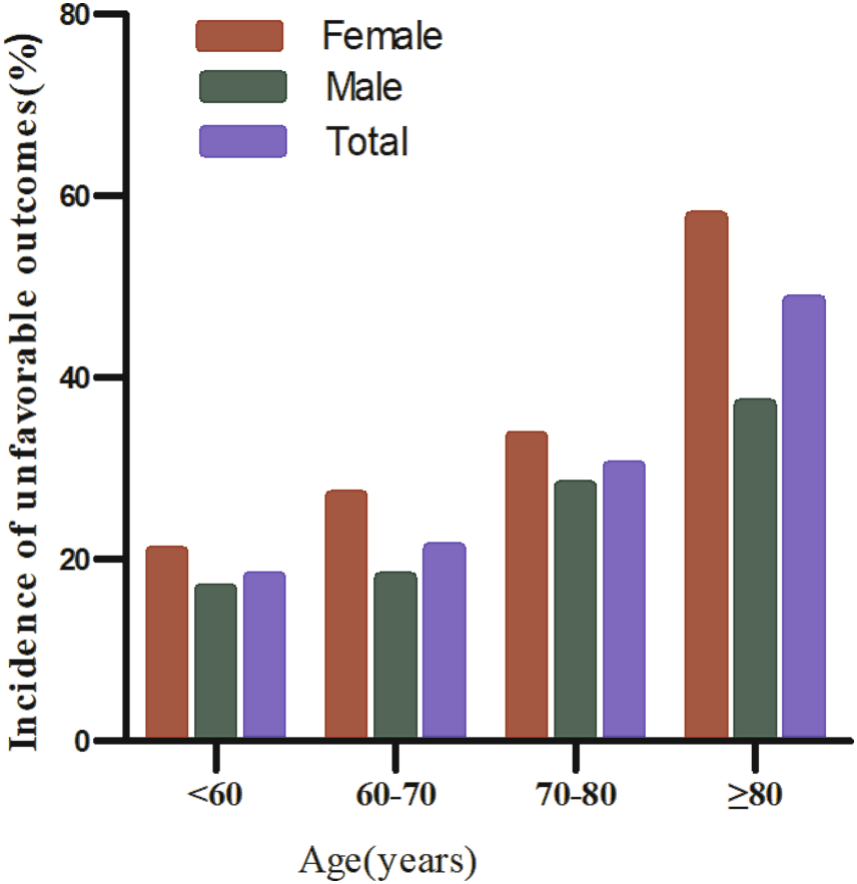
90-day unfavorable outcome incidence in AIS patients stratified by age and sex.

According to the following age stratification: under 60 years, 60–70 years, 70–80 years, and over 80 years, the incidence of unfavorable outcomes among all age groups of women was higher than that of men (Figure 3). Moreover, the incidence of unfavorable outcomes in both males and females increased with age.

### Relationship between TCBI and 90-day unfavorable outcomes in AIS patients

Three binary logistic regression models were employed to examine the association between TCBI and 90-day unfavorable outcomes among AIS patients (Table 3). When TCBI was analyzed as a continuous variable (per 100-unit increment), a consistent inverse association with unfavorable outcomes was observed across all models, although the magnitude of the effect attenuated progressively with increasing covariate adjustment. In the unadjusted Model I, each 100-unit increase in TCBI was associated with a 4.9% reduction in the risk of 90-day unfavorable outcomes (OR = 0.951, 95% CI: 0.936–0.967, *p* < 0.001). After adjusting for age and sex in Model II, the effect size decreased slightly, with each 100-unit increment in TCBI associated with a 3.4% risk reduction (OR = 0.966, 95% CI: 0.950–0.982, *p* < 0.001). This attenuation suggests that demographic factors (age and sex) partially confound the relationship between TCBI and prognosis. In the fully adjusted Model III, which accounted for multiple potential confounders including DM, PLT, sex, AST, age, hypertension, HBA1c, stroke etiology, NIHSS score, HDL-c, AF, smoking, and Scr, the association remained statistically significant: for every 100-unit increase in TCBI, the risk of unfavorable outcomes at 90 days decreased by 2.8% (OR = 0.972, 95% CI: 0.953–0.991, *p* = 0.004). The persistence of this association after comprehensive adjustment indicates that TCBI is independently associated with 90-day functional outcomes in AIS patients, independent of established demographic, metabolic, and clinical risk factors.

**Table 3:** Association of TCBI with 90-day unfavorable outcomes follow AIS in different models.

Exposure	Model I (OR 95%CI) *p*-value	Model II (OR 95%CI) *p*-value	Model III (OR 95%CI) *p*-value	Model IV (OR 95%CI) *p*-value
TCBI (per 100-unit)	0.951 (0.936, 0.967) < 0.001	0.966 (0.950, 0.982) < 0.001	0.972 (0.953, 0.991) 0.004	0.976 (0.957, 0.995) 0.012
TCBI quartiles				
Q1	Ref	Ref	Ref	Ref
Q2	0.914 (0.694, 1.206) 0.526	1.040 (0.782, 1.384) 0.787	1.020 (0.735, 1.415) 0.907	1.016 (0.730, 1.413) 0.926
Q3	0.528 (0.394, 0.709) < 0.001	0.614 (0.453, 0.832) 0.002	0.705 (0.498, 0.999) 0.049	0.713 (0.501, 1.014) 0.060
Q4	0.407 (0.299, 0.554) < 0.001	0.547 (0.397, 0.754) < 0.001	0.634 (0.435, 0.925) 0.018	0.679 (0.465, 0.991) 0.045
P for trend	<0.001	<0.001	0.007	0.013

Model I: No covariates were adjusted. Model II: Age and sex were adjusted. Model III: DM, PLT, sex, AST, age, HTN, HBA1c, stroke etiology, NIHSS score, HDL-c, AF, smoking, and Scr were adjusted. Model IV: DM, PLT (smooth), sex, AST (smooth), age, HTN, HBA1c(smooth), stroke etiology, NIHSS scor e (smooth), HDL-c(smooth), AF, smoking, and Scr(smooth) were adjusted.

Additionally, TCBI was categorized according to its quartiles and entered the fully adjusted logistic regression model (Table 3, Model III). Using the lowest quartile (Q1) as the reference group, a clear graded protective effect of higher TCBI levels was observed (Table 3). After multivariable adjustment, the ORs for 90-day unfavorable outcomes were 1.020 (95% CI: 0.735–1.415, *p* = 0.907) for Q2, 0.705 (95% CI: 0.498–0.999, *p* = 0.049) for Q3, and 0.634 (95% CI: 0.435–0.925, *p* = 0.018) for Q4, compared with Q1. These results indicate that although Q2 did not differ significantly from Q1 in terms of 90-day unfavorable outcomes, Q3 and Q4 exhibited markedly lower risks with statistical significance. Overall, there was a decreasing trend in the risk of 90-day unfavorable outcomes from Q1 to Q4, indicating that higher TCBI levels are associated with better functional prognosis, the test for linear trend was significant (P for trend = 0.007).

### Sensitivity analysis

Multiple sensitivity analyses were performed to determine the robustness of our findings. Initially, the GAM method was applied to include continuous covariates as smooth curves in the model. The results (Table 3, Model IV) closely aligned with the fully adjusted Model III outcomes. Specifically, for every 100 units increase in TCBI, the risk of unfavorable outcomes at 90 days was reduced by 2.4% (OR = 0.976, 95% CI: 0.957–0.995). Further sensitivity analyses demonstrated that the significant negative relationship between TCBI (for every 100 units increase) and the risk of unfavorable outcomes at 90 days remained after excluding patients with DM (OR = 0.969, 95% CI: 0.944–0.993). Similarly, when excluding smokers, for every 100-unit increase in TCBI, the OR for unfavorable outcomes was 0.971 (95% CI: 0.946–0.996). In addition, in the analysis limited to the study population with a BMI less than 28 kg/m^2^, a significant association between TCBI and unfavorable outcomes at 90 days was still present (OR = 0.972, 95% CI: 0.952–0.992). After participants with missing values in any covariates (complete cases) were excluded, a sensitivity analysis was conducted. The results indicated that an OR of 0.964 (95%CI: 0.945, 0.983) was observed for the association between TCBI (per 100-unit) and 90-day unfavorable outcomes, which was consistent with the results from the data analysis after multiple imputations (Table 4).

**Table 4:** Relationship between TCBI and 90-day unfavorable outcomes after AIS across various sensitivity analyses.

Exposure	Model I (OR 95%CI) *p*-value	Model II (OR 95%CI) *p*-value	Model III (OR 95%CI) *p*-value	Model IV (OR 95%CI) *p*-value
TCBI (per 100-unit)	0.969 (0.944, 0.993) 0.013	0.971 (0.946, 0.996) 0.024	0.972 (0.952, 0.992) 0.007	0.964 (0.945, 0.983) < 0.001
TCBI quartiles				
Q1	Ref	Ref	Ref	Ref
Q2	0.939 (0.629, 1.403) 0.759	0.946 (0.632, 1.415) 0.786	0.990 (0.711, 1.380) 0.955	0.884 (0.629, 1.241) 0.476
Q3	0.638 (0.411, 0.989) 0.044	0.699 (0.453, 1.078) 0.105	0.690 (0.482, 0.986) 0.042	0.622 (0.437, 0.886) 0.009
Q4	0.596 (0.368, 0.964) 0.035	0.590 (0.362, 0.963) 0.035	0.637 (0.430, 0.945) 0.025	0.513 (0.353, 0.747) < 0.001
P for trend	0.011	0.016	0.006	<0.001

Model I was a sensitivity analysis in participants without DM (*n* = 1,206). PLT, sex, AST, age, HTN, HBA1c, stroke etiology, NIHSS score, HDL-c, AF, smoking, and Scr were adjusted. Model II was a sensitivity analysis in participants without smoking (*n* = 1,067). DM, PLT, sex, AST, age, HTN, HBA1c, stroke etiology, NIHSS score, HDL-c, AF, and Scr were adjusted. Model III was a sensitivity analysis in participants without BMI ≥28 kg/m^2^ (*n* = 1,637). Age, sex, PLT, D-dimer, Scr, HBA1c, LDL-c, AST, hypertension, CHD, smoking, AF, DM, stroke etiology, and NIHSS score were adjusted. Model IV was a sensitivity analysis using complete case data (before multiple imputation; *n* = 1,560), adjusted for DM, PLT, sex, AST, age, HTN, HBA1c, stroke etiology, smoking, NIHSS score, HDL-c, AF, and Scr. OR, odds ratios; CI, confidence; Ref, reference.

When TCBI was analyzed by quartiles in the sensitivity analyses, a broadly consistent pattern with the main analysis was observed across the different models (Table 4). In patients without DM, compared with Q1, the fully adjusted ORs for 90-day unfavorable outcomes were 0.939 (95% CI 0.629–1.403) for Q2, 0.638 (95% CI 0.411–0.989) for Q3, and 0.596 (95% CI 0.368–0.964) for Q4, with a significant trend (P for trend = 0.011). In those without smoking, the corresponding ORs were 0.946 (95% CI 0.632–1.415) for Q2, 0.699 (95% CI 0.453–1.078) for Q3, and 0.590 (95% CI 0.362–0.963), with P for trend = 0.016. Among participants with BMI < 28 kg/m^2^, the ORs were 0.990 (95% CI 0.711–1.380) for Q2, 0.690 (95% CI 0.482–0.986) for Q3, and 0.637 (95% CI 0.430–0.945) for Q4 (P for trend = 0.006). Although Q2 consistently showed no significant difference from Q1, higher quartiles (Q3 and Q4) were generally associated with lower odds of 90-day unfavorable outcomes after multivariable adjustment, which is consistent with the main findings from Model III in Table 3. The statistically significant trend tests in these sensitivity analyses further demonstrate an overall decreasing pattern in the odds of unfavorable outcomes from Q1 to Q4.

In the sensitivity analysis without excluding extreme TCBI values, the inverse association between TCBI and 90-day unfavorable outcomes after AIS remained significant. After full adjustment for potential confounders(DM, PLT, sex, AST, age, HTN, HBA1c, stroke etiology, NIHSS score, HDL-c, AF, smoking, and Scr), each 100-unit increase in TCBI was associated with an approximately 1.5% reduction in the risk of 90-day unfavorable outcomes (OR = 0.985, 95% CI: 0.972–0.999). When TCBI was analyzed by quartiles, participants in the third and fourth quartiles continued to have a significantly lower risk of 90-day unfavorable outcomes compared with those in the lowest quartile (Q3 vs. Q1: OR = 0.601, 95% CI: 0.440–0.821; Q4 vs. Q1: OR = 0.634, 95% CI: 0.455–0.882). The test for trend likewise indicated a progressively decreasing incidence of unfavorable outcomes from the first to the fourth quartile, further supporting the robustness of this association (Supplementary Table S1).

Furthermore, the computed E-value reached 1.20, surpassing the relative risk for TCBI and potential unmeasured confounders (1.17) while remaining below the relative risk for unmeasured confounders and 90-day unfavorable outcomes (1.24). This finding suggests that unidentified or unconsidered confounding variables are unlikely to have a significant influence on the TCBI-unfavorable outcome relationship. These sensitivity tests additionally strengthened the credibility and validity of our research results.

### Nonlinear association between TCBI and 90-day unfavorable outcomes

**Figure 4:**
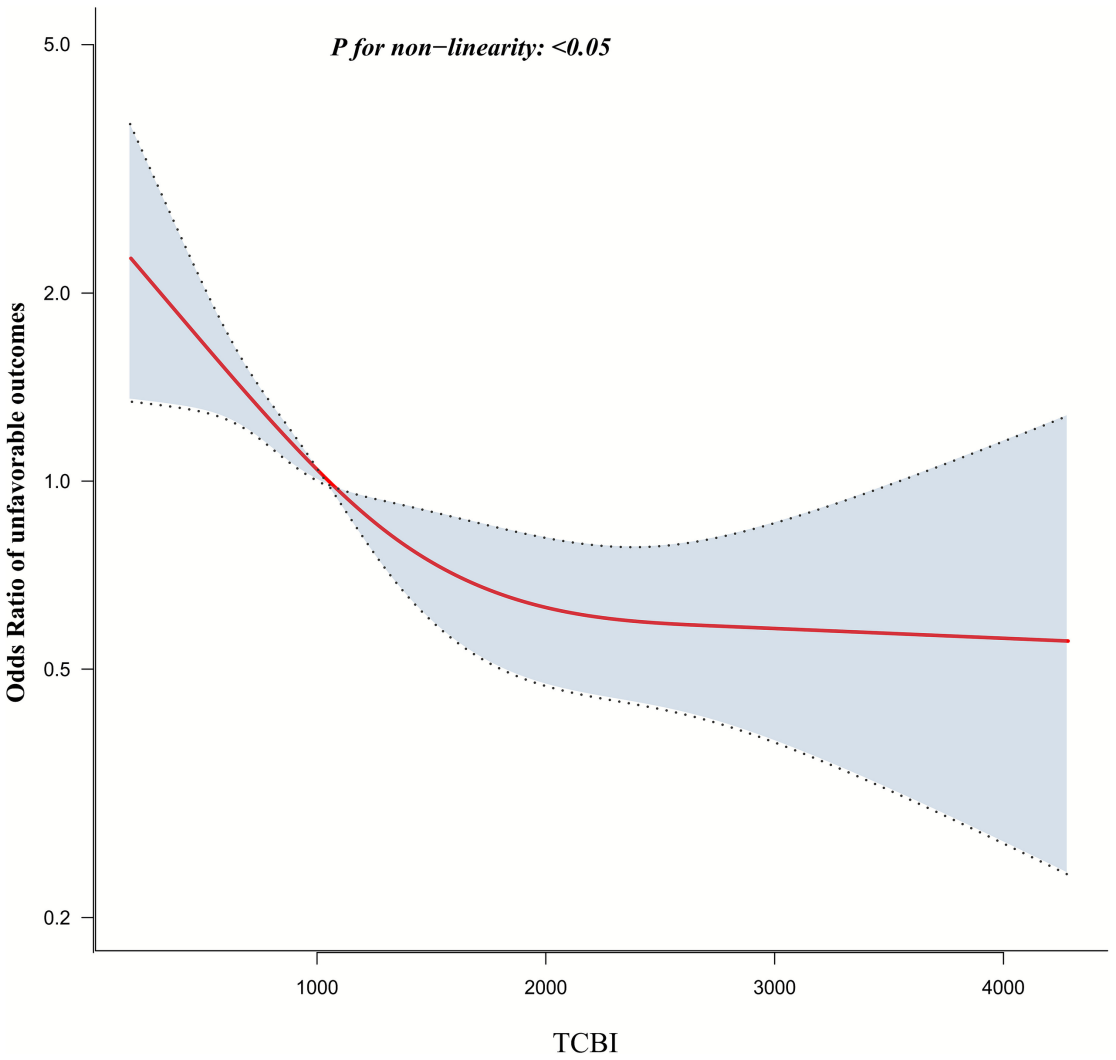
The nonlinear relationship between TCBI and the risk of 90-day unfavorable outcomes in AIS patients.

A logistic regression model incorporating restricted cubic spline functions was used to discover a nonlinear relationship between TCBI and 90-day unfavorable outcomes (p for nonlinearity < 0.05; Figure 4). Recursive analysis identified a turning point at 1227.3 for TCBI. Subsequently, a segmented logistic regression model was employed to calculate the ORs and CIs on either side of the turning point. Below the turning point, the OR linking TCBI (per 100-unit) with 90-day unfavorable outcome risk was 0.928 (95% CI: 0.8866–0.9714). Above this turning point, the OR measured 0.983 (95% CI: 0.955–1.287), failing to achieve statistical significance (Table 5).

**Table 5:** Relationship between TCBI and 90-day unfavorable outcomes analyzed by two-piecewise logistic regression model.

Outcome: 90-day unfavorable outcome	OR (95%CI) *p*-value
Fitting Model by two-piecewise linear regression	
Inflection point of TCBI	1227.3
TCBI <1227.3 (per 100-unit)	0.928 (0.8866, 0.9714) 0.001
TCBI > 1227.3 (per 100-unit)	0.983 (0.955, 1.287) 0.226
P for log-likelihood ratio test	0.047

DM, PLT, sex, AST, HTN, HBA1c, stroke etiology, NIHSS score, HDL-c, AF, smoking, and Scr were adjusted.

### Subgroup analysis

Across all pre-specified or exploratory subgroup analyses (Table 6), no substantial interaction effects were detected between TCBI and variables including hypertension, sex, prior stroke/TIA, age, CHD, stroke etiology, and AF (with all *p*-values ≥ 0.05). These findings imply that these covariates do not influence or alter the relationship between TCBI and 90-day unfavorable outcomes in AIS patients.

**Table 6:** Stratified associations of TCBI with 90-day unfavorable outcomes follow AIS in by previous stroke/TIA, age, stroke etiology, sex, hypertension, CHD, and AF.

Characteristic	Participants	OR (95%CI) *p* value	P for interaction
Age(years)			0.7126
<60	386	0.977 (0.942, 1.014) 0.215	
60–70	470	0.977 (0.941, 1.015) 0.234	
70–80	630	0.955 (0.923, 0.989) 0.009	
≥80	278	0.981 (0.940, 1.025) 0.398	
Sex			0.5530
Male	1,072	0.976 (0.952, 1.000) 0.053	
Female	692	0.965 (0.937, 0.994) 0.019	
Previous stroke/TIA			0.5750
No	1,396	0.971 (0.951, 0.992) 0.008	
Yes	368	0.985 (0.943, 1.028) 0.486	
Hypertension			0.4991
No	644	0.980 (0.950, 1.011) 0.204	
Yes	1,120	0.967 (0.944, 0.990) 0.006	
CHD			0.8927
No	1557	0.971 (0.951, 0.991) 0.005	
Yes	207	0.975 (0.920, 1.033) 0.385	
AF			0.2780
No	1,388	0.967 (0.947, 0.988) 0.002	
Yes	376	0.994 (0.950, 1.040) 0.798	
Stroke etiology (n, %)			0.7681
SVO	572	0.979 (0.950, 1.009) 0.166	
CE	343	0.983 (0.944, 1.024) 0.411	
LAA	450	0.981 (0.939, 1.025) 0.389	
Other determined	149	0.957 (0.907, 1.010) 0.111	
Undetermined	250	0.945 (0.890, 1.004) 0.069	

Above model adjusted for DM, PLT, sex, AST, age, HTN, HBA1c, stroke etiology, NIHSS score, HDL-c, AF, smoking, and Scr. In each case, the Model is not adjusted for the stratification variable. OR, odds ratios; CI, confidence; AF, atrial fibrillation; SVO, Small Vessel Occlusion; TIA, transient ischemic attack; CE, Cardiac Embolism; LAA, Large Artery Atherosclerosis.

### ROC analysis of the predictive value of nutritional indices for 90-day unfavorable outcomes in AIS patients

**Figure 5:**
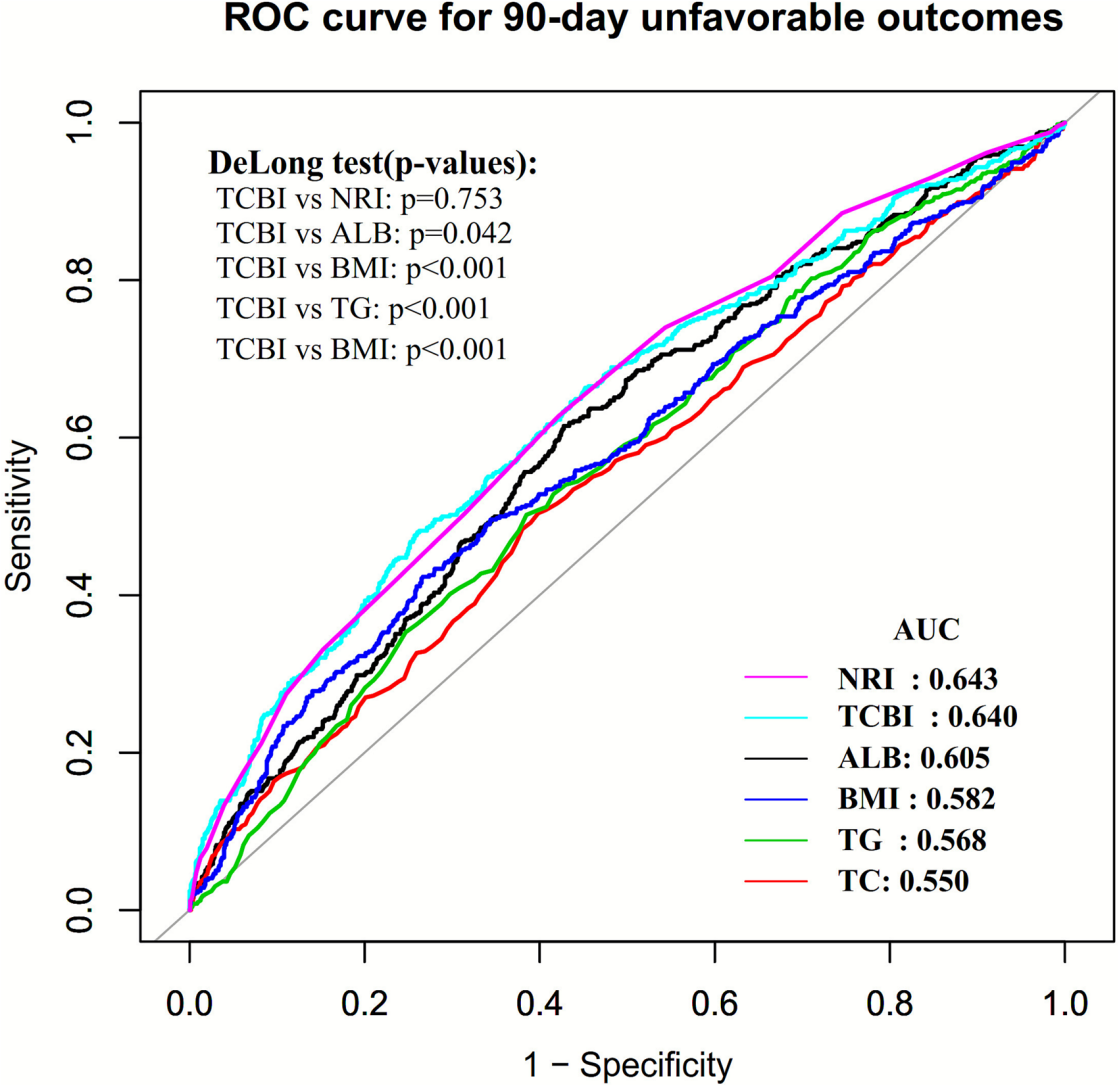
ROC curves of ALB, TC, TG, BMI, NRI, and TCBI for predicting 90-day unfavorable outcomes in patients with AIS.

The ROC curve was plotted to evaluate the discriminative ability of ALB, TC, TG, BMI, NRI, and TCBI for predicting 90-day poor outcomes in patients with AIS (Figure 5). The AUC values for each variable were as follows: TG: 0.5677 (95% CI: 0.5381–0.5973) < TC: 0.5502 (95% CI: 0.5199–0.5806) < BMI: 0.5824 (95% CI: 0.5518–0.6130) < ALB: 0.6048 (95% CI: 0.5756–0.6340) < TCBI: 0.6332 (95% CI: 0.6145–0.6719) < NRI: 0.6397 (95% CI: 0.6103–0.6691). The Youden index values for ALB, TC, TG, BMI, NRI, and TCBI were 0.1875, 0.1057, 0.1171, 0.1568, 0.2195, and 0.2059, respectively. These findings indicate that among the nutritional indices examined, the composite parameters NRI and TCBI have relatively higher Youden index values and AUCs. Further comparison of ROC curves using the DeLong test showed no significant difference between NRI and TCBI in their ability to predict 90-day unfavorable outcomes in patients with AIS (Supplementary Table S2).

## Discussion

This study found an independent inverse association between TCBI and 90-day unfavorable outcomes in patients with AIS. After adjustment for potential confounders, each 100-unit increase in TCBI was associated with an OR of 0.972 (95% CI: 0.953–0.991) for 90-day unfavorable outcomes. GAM and multiple sensitivity analyses yielded similar effect estimates, underscoring the independence and stability of this association. The E-value analysis suggested that potential unmeasured confounders are unlikely to fully explain the relationship between TCBI and 90-day unfavorable outcomes. In addition, a non-linear association was observed, with a turning point at a TCBI value of 1227.3, and different association patterns on either side of this threshold. Below the turning point, each 100-unit increase in TCBI was associated with an OR of 0.928 (95% CI: 0.887–0.971) for 90-day unfavorable outcomes.

TCBI is a composite index reflecting nutritional status and metabolic reserves, which is closely related to the risks of stroke, cardiovascular events, and cognitive impairment, among other conditions (32–34). Moreover, TCBI has shown significant value in predicting the prognosis of various diseases, including acute decompensated heart failure, CHD, aortic dissection, cardiac critical patients requiring mechanical circulatory support, as well as diabetic nephropathy (16, 17, 35, 36). However, research on the relationship between TCBI and stroke prognosis is currently limited and inconsistent. In a study based on the CNSR-III data, which included 9,708 AIS patients, the multivariable model revealed that when TCBI was divided into quartiles, the OR for experiencing adverse functional outcomes (mRS score ≥3), recurrent strokes, and all-cause mortality were 1.47 (95% CI:1.22–1.78), 1.09 (95% CI:0.90–11.32)，and 1.34(95% CI:0.94–1.86) for the lowest TCBI levels compared to the highest (19). Another study from the Hacettepe Neurology Acute Stroke Database involving 299 AIS patients who received IV-tPA treatment (20). The results showed that there was no statistical difference in TCBI levels between the good prognosis group (mRS score: 0–2) and the poor prognosis group (mRS score: 3–6) 3 months after AIS, with TCBI values of 2270.1 ± 1902.1 and 1751.5 ± 1667.8, respectively (*p* = 0.595) (20). Additionally, the TCBI level in the survival group (TCBI:1946 ± 1817.7) was not statistically significantly higher than that in the death group (TCBI:1659.2 ± 893.6) at 3 months after AIS (*p* = 0.521) (20). Our study complements the existing literature by supporting a significant negative association between elevated TCBI levels and unfavorable outcomes within 90 days for AIS patients. Notably, there are differences in the effect sizes reported across different studies, which may be related to differences in the study population, sample size, sex distribution, study duration, and the covariates adjusted in each study. Additionally, unlike previous studies, this study evaluated TCBI as both a categorical variable and a continuous variable, thereby reducing information loss and more accurately quantifying its relationship with prognosis. We conducted a sensitivity analysis on participants with a BMI below 28 kg/m^2^ who had no DM or smoking, to validate the robustness of our findings. The robustness of this association was further supported by our subgroup analyses. Across prespecified subgroups defined by hypertension, sex, prior stroke/TIA, age, CHD, stroke etiology, and AF, the direction of the association between TCBI and 90-day unfavorable outcomes remained generally consistent, even though the magnitude of the effect estimates varied between strata. Importantly, none of the interaction terms reached statistical significance, indicating that the inverse association of higher TCBI with a lower risk of 90-day unfavorable outcomes was broadly homogeneous across these subgroups and that no subgroup exhibited a qualitatively different risk pattern. This consistency strengthens the generalizability of our findings. In summary, clarifying the relationship between TCBI and the prognosis of AIS patients provides new insights for improving stroke recovery and management, ultimately enhancing their health status and quality of life. Additionally, this may encourage clinicians to reassess risk evaluation for stroke prognosis and improve strategies for stroke outcomes.

In addition, ROC analysis showed that TCBI had a prognostic performance for 90-day unfavorable outcomes that were broadly comparable to that of NRI. Although NRI is a well-validated composite nutritional index, its calculation depends on serum albumin as well as information on usual body weight and ideal BMI. By contrast, TCBI is derived solely from routinely and rapidly obtainable parameters (TC, TG, and BMI) and is therefore less affected by acute inflammatory fluctuations and missing historical weight data. In this study, no statistically significant difference was observed between TCBI and NRI in predicting 90-day poor outcomes, suggesting that this simplified index can provide risk assessment information comparable to that of established nutritional scores. Clinically, these findings support the use of TCBI as a convenient, low-cost tool for early nutritional risk stratification in patients with acute ischemic stroke. Because TCBI can be calculated immediately from routine admission laboratory tests, it may help clinicians identify high-risk patients at an early stage, thereby facilitating timely, targeted nutritional assessment and intervention.

Currently, the specific mechanism behind the negative relationship between TCBI and short-term prognosis in AIS patients remains unclear, which may be related to nutritional status. Adequate nutritional status has been shown to promote recovery, reduce the risk of infection, and improve the overall prognosis of stroke patients (37, 38). In contrast, malnutrition adversely affects these processes, increasing the likelihood of complications and prolonging recovery time (39). It is noteworthy that low levels of TG, TC, and BW are indicators of malnutrition (40–42). Furthermore, AIS is a state of metabolic stress that requires a significant amount of energy (43). TG storage can provide excess calories under necessary metabolic stress conditions, and lower TG levels may adversely affect early neural repair in patients with AIS (44, 45). Simultaneously, cholesterol plays an important role in the integrity of cell membranes and the repair of neural cells; low levels of TC may affect the composition of brain lipids and its ability to repair (46–49). Thus, TCBI, which integrates TC, TG, and BW, provides a more comprehensive reflection of the nutritional status of AIS stroke patients, and to some extent, reflects the pathophysiological processes of short-term neural injury and repair in these patients.

The relationship between lipid levels and prognosis in AIS patients is complex. Although elevated cholesterol and TG are recognized risk factors for atherosclerosis, excessively low lipid levels may paradoxically worsen functional prognosis and increase hemorrhagic risk in AIS patients (49, 50). Very low LDL-c and TC impair vascular integrity, affect coagulation function, and hinder neural repair, particularly pronounced in patients receiving intensive statin therapy (49, 50). Statins possess multiple biological effects—anti-inflammatory, endothelial-protective, and neuroprotective actions—that reduce stroke recurrence (51). However, in patients with already low lipid levels (low TCBI values), further lipid reduction may increase bleeding risk and impair neuroplasticity, emphasizing the necessity of individualized management (52–54). TCBI integrates TG, TC, and body weight indicators, providing comprehensive nutritional-metabolic assessment that helps identify patients requiring nutritional support while identifying those at risk due to excessively low lipid levels during statin therapy. Clinically, the management of patients with AIS requires a careful balance between lipid control and nutritional optimization. Patients with low TCBI values may need targeted nutritional support and more cautious lipid-lowering, whereas those with higher TCBI values can generally receive standard statin therapy safely.

In addition, after stratifying participants according to the quartiles of the TCBI, the results of the multivariable adjusted model showed that compared to Q1 of the TCBI, the OR for unfavorable outcomes in Q2, Q3, and Q4 were 1.020, 0.705, and 0.634, respectively. This indicated that the OR values were similar between Q1 and Q2, but showed a significant decreasing trend from Q3 to Q4, suggesting a potential nonlinear relationship between them. Using a logistic regression model incorporating restricted cubic splines, the association between TCBI and 90-day unfavorable outcomes showed an approximately “L-shaped” pattern, with an inflection point at a TCBI of 1227.3. At lower TCBI levels, the curve declined steeply, indicating a marked reduction in the risk of unfavorable outcomes with increasing TCBI, whereas at higher TCBI levels, the curve gradually flattened and tended to approach a plateau. Specifically, below this inflection point, each 100-unit increase in TCBI was associated with a 7.2% reduction in the risk of 90-day unfavorable outcomes. However, above 1227.3, this association was no longer statistically significant. From a clinical perspective, this nonlinear pattern and the identified turning point are of considerable importance for risk stratification and management. The threshold of approximately TCBI ≈ 1227.3 may help identify patients with relatively poor nutritional status. For AIS patients with TCBI < 1227.3, more proactive treatment measures may be necessary, such as enhancing nutritional support, more frequent monitoring, strengthening secondary prevention strategies, and implementing more aggressive rehabilitation strategies to reduce the risk of unfavorable outcomes. In contrast, for patients whose TCBI has already reached or exceeded this level, additional increases in TCBI may confer only limited prognostic benefit, highlighting the need to prioritize nutritional screening and intervention in patients with low TCBI. From a practical standpoint, the clinical applicability of this turning point also deserves consideration. First, TCBI is calculated from three routinely available parameters—TG, TC, and body weight—all of which are standard components of admission assessments in AIS patients. Therefore, no additional laboratory tests or procedures are required, and TCBI can be readily obtained via manual calculation, simple bedside calculators, or automatic integration into electronic medical record systems. Second, the threshold of 1227.3 was statistically derived as the inflection point within this cohort and should primarily be regarded as a data-driven reference value rather than an absolute clinical cut-off. In real-world practice, the use of an approximate rounded threshold (e.g., TCBI ≈ 1,200) might be more feasible for risk stratification, with particular attention paid to patients below this level, in whom closer post-stroke monitoring and timely nutritional or metabolic optimization may be warranted. Third, this cut-off was identified in a single-center Korean cohort with specific demographic and clinical characteristics. Before it can be adopted as a generalized decision threshold in diverse healthcare settings, external validation and potential recalibration across different ethnicities, healthcare systems, and treatment paradigms are required. Collectively, these considerations suggest that while the TCBI turning point provides useful guidance for identifying high-risk patients with suboptimal nutritional reserves, it should currently be interpreted as a flexible tool to inform individualized management rather than as a rigid universal boundary.

This study presents several notable strengths. Initially, we investigated the relationship between TCBI and unfavorable outcomes in AIS patients by treating TCBI through dual analytical approaches: continuous and categorical variables (quartile-based stratification). Such methodology reduced data loss while effectively characterizing their association. Subsequently, multiple imputation methods were utilized to address missing values, thereby improving statistical power and reducing potential bias from incomplete covariate data. Moreover, various sensitivity tests were performed to strengthen result reliability, including transforming independent variables, calculating E-values for evaluating unmeasured and unknown confounder effects, and reassessing TCBI-unfavorable outcome associations after excluding participants with BMI ≥ 28 kg/m^2^, smoking, and DM.

Several constraints in this investigation merit consideration. First, the study population in this study was limited to Korean individuals, which may restrict the generalizability of the findings, and external validation in multi-ethnic cohorts is warranted in the future. Second, TCBI alongside other clinical parameters were obtained solely at enrollment, with temporal variations in TCBI remaining unexplored regarding their prognostic implications for AIS patients. This represents a crucial avenue for subsequent investigations, targeting comprehensive data collection encompassing longitudinal TCBI fluctuations. Additionally, certain variables exhibited incomplete information characteristics. Age stratification within the original dataset employed 10 categorical intervals rather than precise chronological values, potentially compromising variable completeness. Future study designs should prioritize collection of more granular variable data. Third, as an observational study, missing data is inevitable. The present study also faced the issue of missing covariates. Although multiple imputations were employed to handle missing values, potential bias may still exist in the results. However, we conducted a sensitivity analysis based on the complete dataset. The results demonstrated that the conclusions remained consistent before and after imputation. Future prospective studies are planned to optimize data collection procedures and reduce the incidence of missing values. Fourth, participants with abnormal and extreme TCBI values (≥3 SD from the mean) were excluded, which may have reduced the representativeness of the study population and led to an underestimation of the prognostic impact of very low or very high TCBI levels. However, a sensitivity analysis including all participants without outlier exclusion yielded similar results, suggesting that this limitation is unlikely to have materially affected our main findings. Concurrently, consistent with most observational investigations, despite controlling for recognized potential confounders, this analysis may harbor unmeasured or uncontrolled confounding variables. Moreover, given that this secondary analysis utilized previously published datasets, adjustment for variables absent from original data collection (such as IV-tPA or endovascular intervention information) proved unfeasible. However, E-value computations indicated that such confounding factors were unlikely to substantially influence study outcomes. Finally, this observational investigation demonstrated independent associations between TCBI and short-term prognosis in AIS patients, yet failed to determine causal relationships between these parameters.

## Conclusion

This study revealed an independent negative association between TCBI and 90-day unfavorable outcomes in AIS patients. A saturation effect curve was observed, with an inflection point at 1227.3. When TCBI was below this threshold, each 100-unit increase was associated with a 7.2% reduction in the risk of unfavorable outcomes. As a simple, economical, and easily obtainable index, TCBI may serve as a useful reference for prognosis stratification, rehabilitation optimization, and clinical management in AIS patients. Future prospective studies are needed to assess whether TCBI-guided strategies can improve the prognosis of AIS patients.

## Data Availability

The datasets presented in this study can be found in online repositories. The names of the repository/repositories and accession number(s) can be found in the article/Supplementary material. The data can be downloaded from Kang et al. (21), https://journals.plos.org/plosone/article?id=10.1371/journal.pone.0228738.

## Glossary

PLT: platelets
Hs-CRP: high-sensitivity C-reactive protein
ALT: alanine aminotransferase
TIA: transient ischemic attack
HDL-c: high-density lipoprotein cholesterol
BUN: blood urea nitrogen
CE: Cardiac Embolism
TCBI: triglyceride-total cholesterol-body weight index
RDW: red cell distribution width
FIB: fibrinogen
NIHSS: National Institutes of Health Stroke Scale
TC: total cholesterol
AF: atrial fibrillation
HGB: hemoglobin
LAA: Large Artery Atherosclerosis
Scr: serum creatinine
DM: diabetes mellitus
TG: triglycerides
AST: aspartate aminotransferase
WBC: white blood cell
HBA1c: hemoglobin A1c
SVO: Small Vessel Occlusion
eGFR: estimated glomerular filtration rate
FPG: fasting plasma glucose
ALB: albumin
LDL-c: low-density lipoprotein cholesterol
BW: body weight
