# Supplementary material for: Association of a novel nutritional marker, the triglyceride-cholesterol-body weight index, with 90-day unfavorable outcomes in acute ischemic stroke: a prospective cohort study
Source: Front Nutr. 2026 Jan 6;12:1707231. doi: 10.3389/fnut.2025.1707231 (PMC12815849; doi:10.3389/fnut.2025.1707231)
Supplement: Supplementary file 1 [file Table_1.DOCX]

Table S1. Association between TCBI and 90‑day unfavorable outcomes after AIS in participants without trimming extreme TCBI values.

| Exposure | Model I(OR 95%CI) p-value | Model II(OR 95%CI) p-value | Model III(OR 95%CI) p-value |
| --- | --- | --- | --- |
| TCBI (per 100-unit) | 0.968 (0.956, 0.981) <0.001 | 0.982 (0.969, 0.995) 0.008 | 0.985 (0.972, 0.999) 0.033 |
| TCBI quartiles |  |  |  |
| Q1 | Ref | Ref | Ref |
| Q2 | 0.893 (0.679, 1.174) 0.416 | 1.031 (0.776, 1.370) 0.832 | 1.056 (0.790, 1.411) 0.714 |
| Q3 | 0.506 (0.378, 0.679) <0.001 | 0.593 (0.438, 0.803) <0.001 | 0.601 (0.440, 0.821) 0.001 |
| Q4 | 0.418 (0.309, 0.566) <0.001 | 0.582 (0.423, 0.800) <0.001 | 0.634 (0.455, 0.882) 0.007 |
| P for trend | <0.001 | <0.001 | <0.001 |

Model I: No covariates were adjusted.

Model II: Age and sex were adjusted.

Model III: DM, PLT, sex, AST, age, HTN, HBA1c, stroke etiology, NIHSS score, HDL-c, AF, smoking, and Scr were adjusted.

Table S2 Predictive ability of ALB, TC, TG, BMI, NRI, and TCBI for 90‑day unfavorable outcomes in patients with AIS

| Test | AUC (95% CI) | Best threshold | Specificity | Sensitivity | Youden index |
| --- | --- | --- | --- | --- | --- |
| ALB (g/dL) | 0.6048 (0.5756–0.6340) | 40.500 | 0.5726 | 0.6149 | 0.1875 |
| TC (mg/dL) | 0.5502 (0.5199–0.5806) | 169.500 | 0.6017 | 0.5040 | 0.1057 |
| TG (mg/dL) | 0.5677 (0.5381–0.5973) | 88.500 | 0.6151 | 0.5020 | 0.1171 |
| BMI (kg/m²) | 0.5824 (0.5518–0.6130) | 21.877 | 0.7334 | 0.4234 | 0.1568 |
| NRI | 0.6397 (0.6103–0.6691) | 101.833 | 0.7397 | 0.4798 | 0.2195 |
| TCBI | 0.6332 (0.6145–0.6719) | 1012.06 | 0.5789 | 0.6270 | 0.2059 |
